# Factors influencing fall-related serious injury in patients with poststroke: A multicenter cross-sectional study in China

**DOI:** 10.1097/MD.0000000000045661

**Published:** 2025-11-07

**Authors:** Yucong Zou, Fubing Zha, Jing Zhou, Weiming Lin, Yulong Wang

**Affiliations:** a Department of Rehabilitation, Shenzhen Second People’s Hospital, The First Affiliated Hospital of Shenzhen University, Shenzhen, China; b Department of Rehabilitation, Longhua People’s Hospital of Shenzhen, Shenzhen, China.

**Keywords:** Barthel index, factors, fall-related serious injury, Longshi scale, stroke

## Abstract

Patients with stroke frequently encounter complications such as falls, which can adversely affect their functional recovery and exacerbate the overall burden of poststroke rehabilitation. However, the factors affecting fall-related serious injury remain unclear. The objective of the study was to explore the factors influencing fall-related serious injury in patients with poststroke. We included 7430 patients with poststroke. We collected demographic, sociological, and clinical data and assessed activities of daily living using the Barthel Index and the Longshi Scale. Fall-related injury was categorized as either “none/mild” or “severe.” Univariate and multivariate logistic regression models were used to assess influencing factors. Out of 7430 poststroke patients, 624 experienced falls, show the incidence rate of 8.40%. Among the patients who fell, 498 experienced no or mild injury, while 126 suffered severe injury, with a serious injury incidence rate of 20.19%. 498 experienced no/mild fall-related injury, while 126 had severe fall-related injury. In adjusted multivariate analyses, age, living situation, residential floor, the Barthel Index’s bed-chair transfer item score, and the Longshi Scale grade independently influenced fall-related serious injury. This study revealed factors influencing fall-related serious injury in patients with poststroke, providing valuable insights for comprehensive fall management strategies.

## 1. Introduction

Stroke can lead to a range of complications, and falls are a common occurrence poststroke.^[1]^ The incidence of falls in patients with stroke has been reported to be as high as 36%.^[2,3]^ Falls in stroke patients can result in severe injuries such as fractures, subdural hematomas, and even death.^[4]^ Injurious falls not only increase hospitalization costs but also potentially extend the patient’s hospital stay by approximately 1 week.^[5,6]^ The multifaceted and profound consequences of fall-related serious injury have become a growing concern for both clinicians and nurses.^[7]^ Therefore, preventing fall-related serious injury in patients with stroke is important.

Understanding the factors that influence the severity of fall-related injuries is essential for implementing effective preventive interventions.^[8]^ Several studies have indicated various factors for fall-related serious injury, including demographic characteristics, individual patient differences, and environmental conditions.^[8–10]^ The likelihood of fall and fall-related injury increases with the accumulation of risk factors.^[11,12]^ Age is considered a major risk factor for injurious falls, with numerous studies demonstrating that the risk increases with advancing age.^[9]^ A history of prior falls, stroke, and the use of psychotropic medications also significantly elevate the risk.^[8,13]^ Environmental factors – such as falls occurring outside the ward, in bathrooms, or on wet surfaces – are particularly hazardous.^[9,14]^ In addition, activities of daily living (ADL) have also been associated with injurious falls.^[15,16]^ For patients with stroke, the consequences of fall-related injuries may be more severe.^[13]^ Although not all falls result in injury, stroke patients who experience severe fall-related injuries are more likely to suffer functional decline and compromised rehabilitation outcomes.^[12,17]^ However, the factors influencing the fall-related serious injury in poststroke patients remain unclear.

Therefore, the purpose of this study was to investigate the factors that influence fall-related injury in patients with poststroke. The findings may help inform targeted prevention strategies and provide evidence to support effective fall management in patients with poststroke.

## 2. Methods

### 2.1. Study design

This was a multicenter cross-sectional study conducted by the rehabilitation departments of 103 hospitals across 23 Chinese cities from September 2018 to April 2020. This study was approved by the Ethics Committee of the Shenzhen Second People’s Hospital (#20180926006) and registered in the Chinese Clinical Trial Registry (ChiCTR-2000034067). The study follows the principles of the Declaration of Helsinki. Written informed consent was obtained from all patients or their proxies.

### 2.2. Participants

A total of 7430 patients with stroke were recruited from 103 hospitals, including both outpatient and inpatient centers. Stroke diagnosis was based on the International Classification of Diseases, 10th revision (hemorrhagic subtypes: I60.x and I61.x; ischemic subtypes: H34.1, I63.x, and I64.x).^[18]^ The inclusion criteria for this study were as follows: diagnosed with stroke, aged 18 years or older, and experienced a fall event poststroke onset. Conversely, the exclusion criteria were as follows: had not experienced a fall after stroke onset, inability to read or answer questions, aphasia and mental illnesses, mini-mental state examination scores <27 for poststroke patients who had cognitive impairment were excluded. This was since patients with mini-mental state examination scores <27 were unable to complete subsequent ADL assessments.

**Figure 1.**
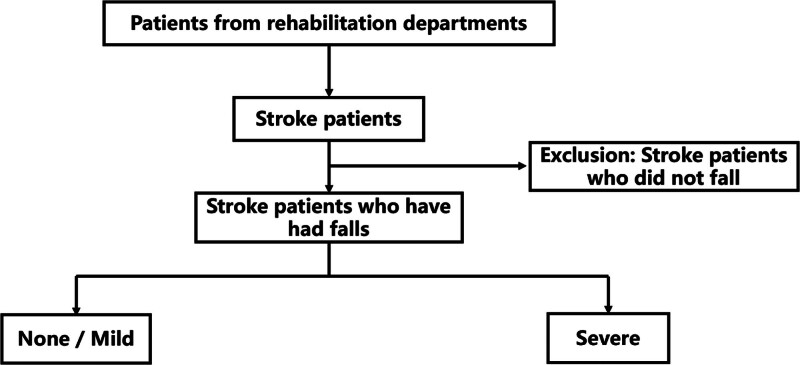
Flow chart of study process.

Based on the inclusion and exclusion criteria, 624 patients with stroke were included in this study. Written informed consent was obtained from all patients or their proxies. The study procedure is illustrated in Figure 1.

### 2.3. Data collection

#### 2.3.1. Demographic and sociology information

A questionnaire survey was used to collect the patients’ demographic data. Demographic characteristics included age, sex, and marital status. Sociological information included living situation, family income, residential floor, elevator usage, and social participation.

Fall incidence was assessed through a single-item question: “Have you experienced any falls in the past 3 months, and if so, what was the outcome?”^[19]^ Fall-related injury was classified into 2 categories: “none/mild” and “severe,” after considering the National Database of Nursing Quality Indicators categories and clinical manifestations.^[20]^ Specifically, falls resulting in bruises, abrasions, and minor lacerations were defined as “mild” while those leading to fractures, head injuries, and lacerations requiring sutures were defined as “severe.”

#### 2.3.2. Clinical information

Clinical information was obtained automatically from the patients’ e-medical records, including primary diagnosis, hypertension, hyperlipidemia, diabetes, cardiology, illness duration, number of episodes, drinking history, and psychotropic drug use history.

#### 2.3.3. ADL assessment

The participants’ ADL were evaluated with the Barthel Index (BI) and the Longshi Scale (LS). The BI is a widely used scale for ADL evaluation and has demonstrated reliability and validity in assessing outcomes in patients with stroke.^[21,22]^ The BI comprises 10 items, including: feeding, self-bathing, personal toilet, dressing, controlling bowel movements, bladder control, getting on and off the toilet, bed-chair transfer, walking on a level surface, and ascending and descending stairs.^[23]^ Among these items listed, the bed-chair transfer item was selected for analysis, as it was considered a valid indicator of the patient’s limb strength and balance.^[24,25]^

**Figure 2.**
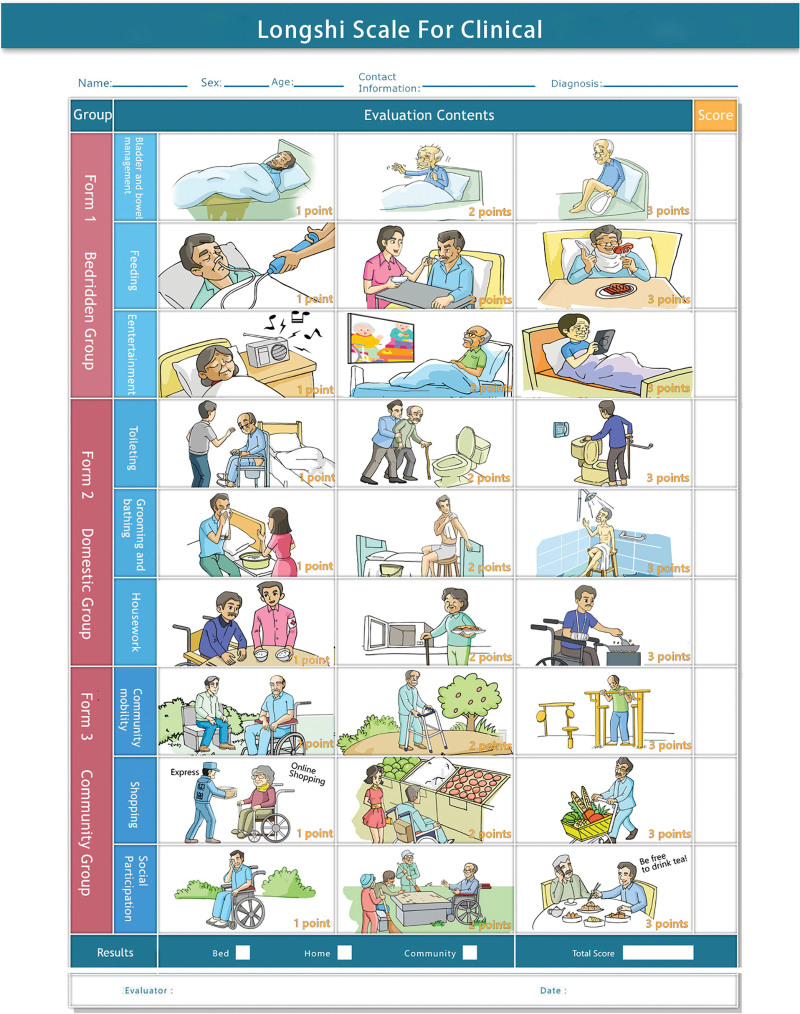
The Longshi scale.

**Figure 3.**
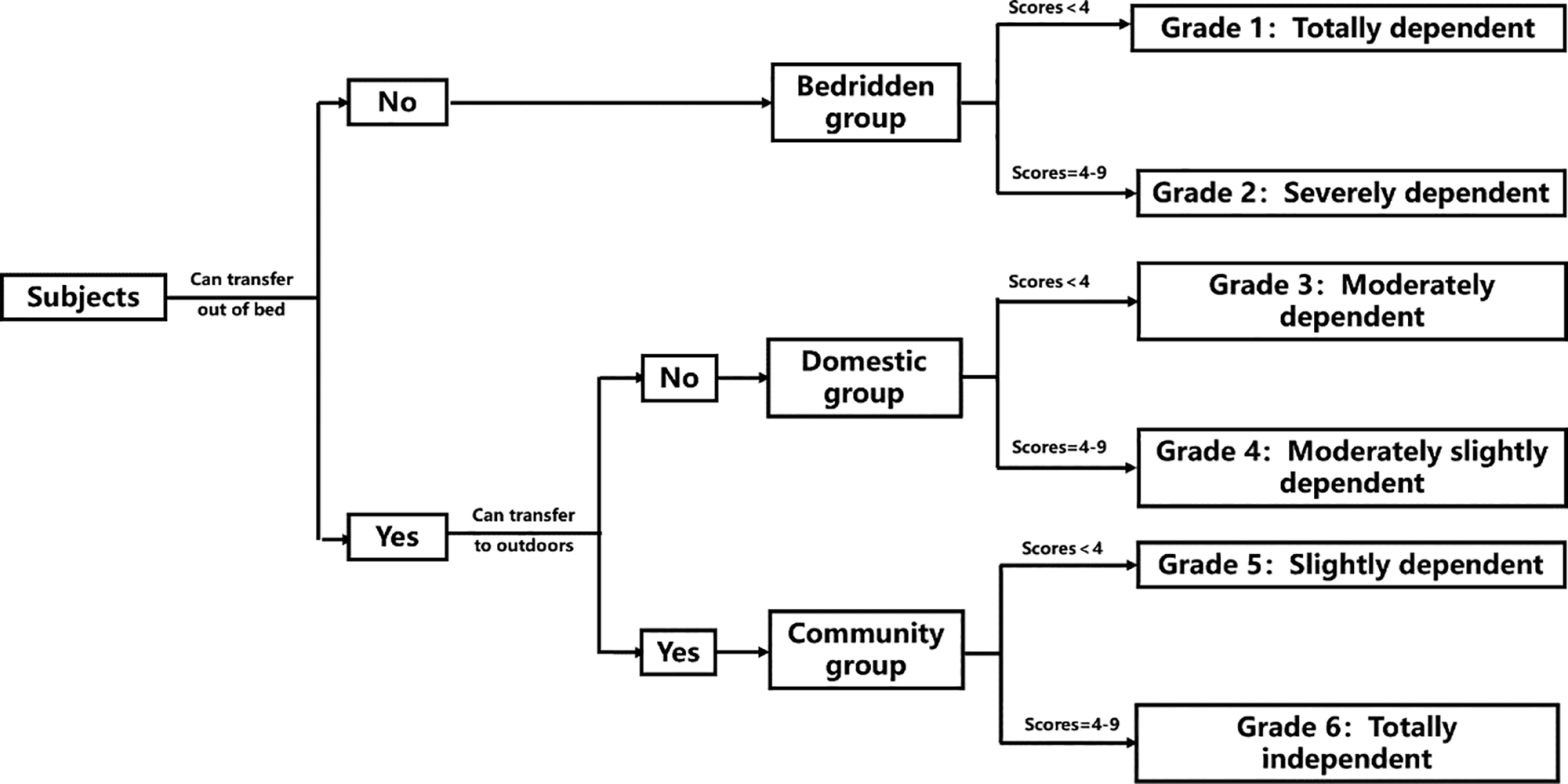
Flowchart for the evaluation of the Longshi scale.

The LS is a novel, image-based tool that was designed by the Rehabilitation Department of Shenzhen Second People’s Hospital to evaluate the ADL of patients with functional disabilities (Fig. 2).^[26]^ It has good reliability and validity and has achieved recognition as one of China national standards for evaluating functional independence and disability (license code: GB/T 37103-2018).^[23,26,27]^ As shown in Figure 3, the LS first categorized patients into either bedridden or non-bedridden groups based on their ability to independently get out of bed. Subsequently, the non-bedridden group was further subdivided into domestic or community groups, depending on their ability to independently engage in outdoor activities. Finally, the LS subscale assessed patients across 3 subitems. The bedridden group was evaluated for bladder and bowel management, feeding, and leisure activities. The domestic group was assessed for toileting, grooming and bathing, and housework. The community group was evaluated for community mobility, shopping, and social participation. The score for each item varies from 1 to 3, and the total score varies from 3 to 9. Based on the subgrouping and total scores, the ADL of patients can be classified into 6 grades: Grade 1 represents complete dependence, Grade 2 indicates severe dependence, Grade 3 signifies moderate dependence, Grade 4 represents slight to moderate dependence, Grade 5 indicates slight dependence, and Grade 6 represents complete independence (Fig. 3).^[26]^

In our study, the BI and LS were assessed by registered clinicians, nurses, or therapists using a patient management application. Patients were randomly assigned to assessors, and their assessments were conducted face-to-face. All data were recorded using electronic spreadsheets.

### 2.4. Data analysis

EmpowerStats software (www.empowerstats.com, X&Y Solutions, Inc., Boston, MA, USA) and R (http://www.R-project.org) were used for data analysis. Continuous variables are presented as mean ± standard deviation when the data followed a normal distribution, and as median or interquartile range when the data had a skewed distribution. Categorical variables are presented as frequencies and percentages. All statistical hypothesis tests were 2-tailed, and *P* <.05 was considered significant.

We first conducted baseline comparisons of demographic and clinical characteristics between the 2 fall-severity groups. Differences between the 2 groups were examined using the Wilcoxon rank-sum test, 2-sample t-test, and χ^2^ test. We explored the influencing factors of fall-related serious injury in patients with stroke using both univariate and multivariate logistics regression analyses.

## 3. Results

### 3.1. Baseline characteristics

Among 7430 poststroke patients, a total of 624 experienced falls, resulting in an incidence rate of 8.40%. Among the patients who fall, 498 experienced no or mild fall-related injury, while 126 suffered severe fall-related injury. The incidence rate of fall-related serious injury among poststroke patients after fall was 20.19%.

Table 1 shows the baseline characteristics of the 624 patients included in the study. Demographically, more than half patients were male (n = 364, 58.33%), older than 60 years (n = 430, 68.91%), and married (n = 537, 86.06%). Living situation, family income, residential floor, elevator usage, and social participation all showed significant differences between the 2 fall-related serious injury groups. Furthermore, most patients had 1 episode (n = 456, 73.08%) and a disease duration of <6 months (n = 488, 83.99%).

**Table 1:** The baseline characteristics.

Variables	N (%)	Severity of fall		*P*-value
		None/mild (%)	Severe (%)	
Total	624 (100%)	498 (79.81%)	126 (20.19%)	–
Age				
<60 years	194 (31.09%)	173 (34.74%)	21 (16.67%)	<**.001**
≥60 years	430 (68.91%)	325 (65.26%)	105 (83.33%)	
Gender				
Male	364 (58.33%)	297 (59.64%)	67 (53.17%)	.189
Female	260 (41.67%)	201 (40.36%)	59 (46.83%)	
Marital status				
Married	537 (86.06%)	429 (86.14%)	108 (85.71%)	**.032**
Divorce	12 (1.92%)	12 (2.41%)	0 (0.00%)	
Widowed	63 (10.10%)	45 (9.04%)	18 (14.29%)	
Unmarried	12 (1.92%)	12 (2.41%)	0 (0.00%)	
Residence status				
Living with others	583 (93.43%)	459 (92.17%)	124 (98.41%)	**.011**
Living alone	41 (6.57%)	39 (7.83%)	2 (1.59%)	
Family income				
<50,000 ¥ (< 6976.52 $)	273 (43.89%)	229 (46.17%)	44 (34.92%)	**.027**
50,000–200,000 ¥ (6976.52–27,906.07 $)	319 (51.29%)	241 (48.59%)	78 (61.90%)	
>200,000 ¥ (> 27,906.07 $)	30 (4.82%)	26 (5.24%)	4 (3.17%)	
Diagnostic				
Cerebral hemorrhage	166 (26.60%)	136 (27.31%)	30 (23.81%)	.427
Cerebral infarction	458 (73.40%)	362 (72.69%)	96 (76.19%)	
Duration of the disease				
<6 months	488 (83.99%)	393 (84.70%)	95 (81.20%)	.356
≥6 months	93 (16.01%)	71 (15.30%)	22 (18.80%)	
Number of episodes				
1	456 (73.08%)	375 (75.30%)	81 (64.29%)	**.026**
2	128 (20.51%)	96 (19.28%)	32 (25.40%)	
>2	40 (6.41%)	27 (5.42%)	13 (10.32%)	
Hypertensive				
No	176 (28.25%)	134 (26.96%)	42 (33.33%)	.156
Yes	447 (71.75%)	363 (73.04%)	84 (66.67%)	
Diabetes				
No	458 (73.52%)	365 (73.44%)	93 (73.81%)	.933
Yes	165 (26.48%)	132 (26.56%)	33 (26.19%)	
Hyperlipidemia				
No	536 (86.17%)	426 (85.89%)	110 (87.30%)	.681
Yes	86 (13.83%)	70 (14.11%)	16 (12.70%)	
Heart diseases				
No	474 (75.96%)	386 (77.51%)	88 (69.84%)	.072
Yes	150 (24.04%)	112 (22.49%)	38 (30.16%)	
Psychotropic drugs				
No	554 (89.79%)	442 (89.66%)	112 (90.32%)	.826
Yes	63 (10.21%)	51 (10.34%)	12 (9.68%)	
Life satisfaction				
No	437 (72.23%)	342 (70.95%)	95 (77.24%)	.165
Yes	168 (27.77%)	140 (29.05%)	28 (22.76%)	
Floors of residence				
1–4 floors	410 (65.81%)	340 (68.41%)	70 (55.56%)	**.011**
5–10 floors	164 (26.32%)	124 (24.95%)	40 (31.75%)	
>10 floors	49 (7.87%)	33 (6.64%)	16 (12.70%)	
Availability of elevator				
No	413 (66.29%)	339 (68.21%)	74 (58.73%)	**.044**
Yes	210 (33.71%)	158 (31.79%)	52 (41.27%)	
Social participation				
No	376 (60.35%)	287 (57.75%)	89 (70.63%)	**.008**
Yes	247 (39.65%)	210 (42.25%)	37 (29.37%)	
Bed-chair transfer item score of the Barthel Index				
0	148 (24.63%)	88 (18.33%)	60 (49.59%)	**<.001**
5	106 (17.64%)	79 (16.46%)	27 (22.31%)	
10	197 (32.78%)	176 (36.67%)	21 (17.36%)	
15	150 (24.96%)	137 (28.54%)	13 (10.74%)	
Longshi scale grades				
1	59 (9.83%)	38 (7.93%)	21 (17.36%)	**<.001**
2	214 (35.67%)	146 (30.48%)	68 (56.20%)	
3	39 (6.50%)	33 (6.89%)	6 (4.96%)	
4	91 (15.17%)	78 (16.28%)	13 (10.74%)	
5	141 (23.50%)	131 (27.35%)	10 (8.26%)	
6	56 (9.33%)	53 (11.06%)	3 (2.48%)	

Bold values indicate statistical significance (*P*-value < .05).

### 3.2. Univariate analysis

To explore the factors influencing the incidence of serious fall-related injury in patients with poststroke, a univariate analysis was conducted (Table 2). The results showed that age, living situation, family income, number of episodes, residential floor, elevator usage, social participation, BI’s bed-chair transfer item scores, and LS grades were associated with fall-related injury in patients with stroke.

**Table 2:** The results of univariate analysis.

Variables	OR	(95% CI)	*P*-value
Age			
<60 yr	1.0	–	–
≥60 yr	2.66	(1.61–4.40)	**.0001**
Gender			
Male	1.0	–	–
Female	1.30	(0.88–1.93)	.1892
Marital status			
Married	1.0	–	–
Divorce	0.00	(0.00–Inf)	.9825
Widowed	1.59	(0.88–2.85)	.1214
Unmarried	0.00	(0.00–Inf)	.9825
Residence status			
Living with others	1.0	–	–
Living alone	0.19	(0.05–0.80)	**.0232**
Family income			
<50,000 ¥ (<6976.52 $)	1.0	–	–
50,000–200,000 ¥ (6976.52–27,906.07 $)	1.68	(1.12–2.54)	**.0130**
>200,000 ¥ (>27,906.07 $)	0.80	(0.27–2.41)	.6923
Diagnostic			
Cerebral hemorrhage	1.0	–	–
Cerebral infarction	1.20	(0.76–1.89)	.4275
Duration of the disease			
<6 mo	1.0	–	–
≥6 mo	1.28	(0.76–2.17)	.3568
Number of episodes			
1	1.0	–	–
2	1.54	(0.97–2.46)	.0684
>2	2.23	(1.10–4.51)	**.0256**
Hypertensive			
No	1.0	–	–
Yes	0.74	(0.49–1.12)	.1569
Diabetes			
No	1.0	–	–
Yes	0.98	(0.63–1.53)	.9332
Hyperlipidemia			
No	1.0	–	–
Yes	0.89	(0.49–1.58)	.6814
Heart diseases			
No	1.0	–	–
Yes	1.49	(0.96–2.30)	.0731
Psychotropic drugs			
No	1.0	–	–
Yes	0.93	(0.48–1.80)	.8264
Life satisfaction			
No	1.0	–	–
Yes	0.72	(0.45–1.15)	.1662
Floors of residence			
1–4 floors	1.0	–	–
5–10 floors	1.57	(1.01–2.43)	**.0452**
>10 floors	2.35	(1.23–4.51)	**.0098**
Availability of elevator			
No	1.0	–	–
Yes	1.51	(1.01–2.25)	**.0452**
Social participation			
No	1.0	–	–
Yes	0.57	(0.37–0.87)	**.0088**
Bed-chair transfer item score of the Barthel index			
0	1.0	–	–
5	0.50	(0.29–0.87)	**.0132**
10	0.17	(0.10–0.31)	**<.001**
15	0.14	(0.07–0.27)	**<.001**
Longshi scale grades			
1	1.0	–	–
2	0.84	(0.46–1.54)	.5799
3	0.33	(0.12–0.91)	**.0327**
4	0.30	(0.14–0.67)	**.0030**
5	0.14	(0.06–0.32)	**<.001**
6	0.10	(0.03–0.37)	**.0005**

Bold values indicate statistical significance (*P*-value < .05).

Regarding demographics, the risk of severe fall-related injuries was 2.66-fold higher in patients over 60 years compared to patients under 60 years (odds ratio [OR] = 2.66, 95% confidence interval [CI]:1.61–4.40, *P* = .0001). In terms of sociological factors, living alone was a protective factor against severe falls compared to living with others (OR = 0.19, 95% CI: 0.05–0.80, *P* = .0232). Family income and residential floor were positively associated with higher fall-related serious injury. Specifically, patients with a household income of 50,000 to 200,000¥(6976.52–27,906.07 $) had a 1.68-fold increased risk of severe falls compared to those with an income of <50,000 (OR = 1.68, 95% CI: 1.12–2.54, *P* = .0130). Patients living on higher residential floors had an increased risk of severe falls compared to those living on lower floors (OR = 1.57, 95% CI: 1.01–2.43, *P* = .0452; OR = 2.35, 95% CI: 1.23–4.51, *P* = .0098). In addition, elevator usage and social participation also impacted fall-related injury, with the presence of an elevator emerging as a risk factor for fall-related serious injury and participation in social activities emerging as a protective factor (OR = 1.51, 95% CI: 1.01–2.25, *P* = .0452; OR = 0.57, 95% CI: 0.37–0.87, *P* = .0088).

Regarding clinical information, we found that disease duration and number of episodes were positively associated with fall-related serious injury. Patients with more than 2 stroke episodes faced a significantly higher risk of fall-related serious injury (OR = 2.23, 95% CI: 1.10–4.51, *P* = .0256). The bed-chair transfer item score of the BI was a protective factor for fall-related serious injury, with higher scores associated with a lower risk (OR = 0.50, 95% CI: 0.29–0.87, *P* = .0132; OR = 0.17, 95% CI: 0.10–0.31, *P* <.0001; OR = 0.14, 95% CI: 0.07–0.27, *P* <.0001; for scores 5, 10, and 15, respectively). Despite no significant difference between LS grades 1 and 2, higher LS grades were associated with a lower fall-related serious injury risk (OR = 0.33, 95% CI: 0.12–0.91, *P* = .0327; OR = 0.30, 95% CI: 0.14–0.67, *P* = .0030; OR = 0.14, 95% CI: 0.06–0.32, *P* <.0001; OR = 0.10, 95% CI: 0.03–0.37, *P* <.0005; for grades 3, 4, 5, and 6, respectively).

### 3.3. Multivariate analysis

**Figure 4.**
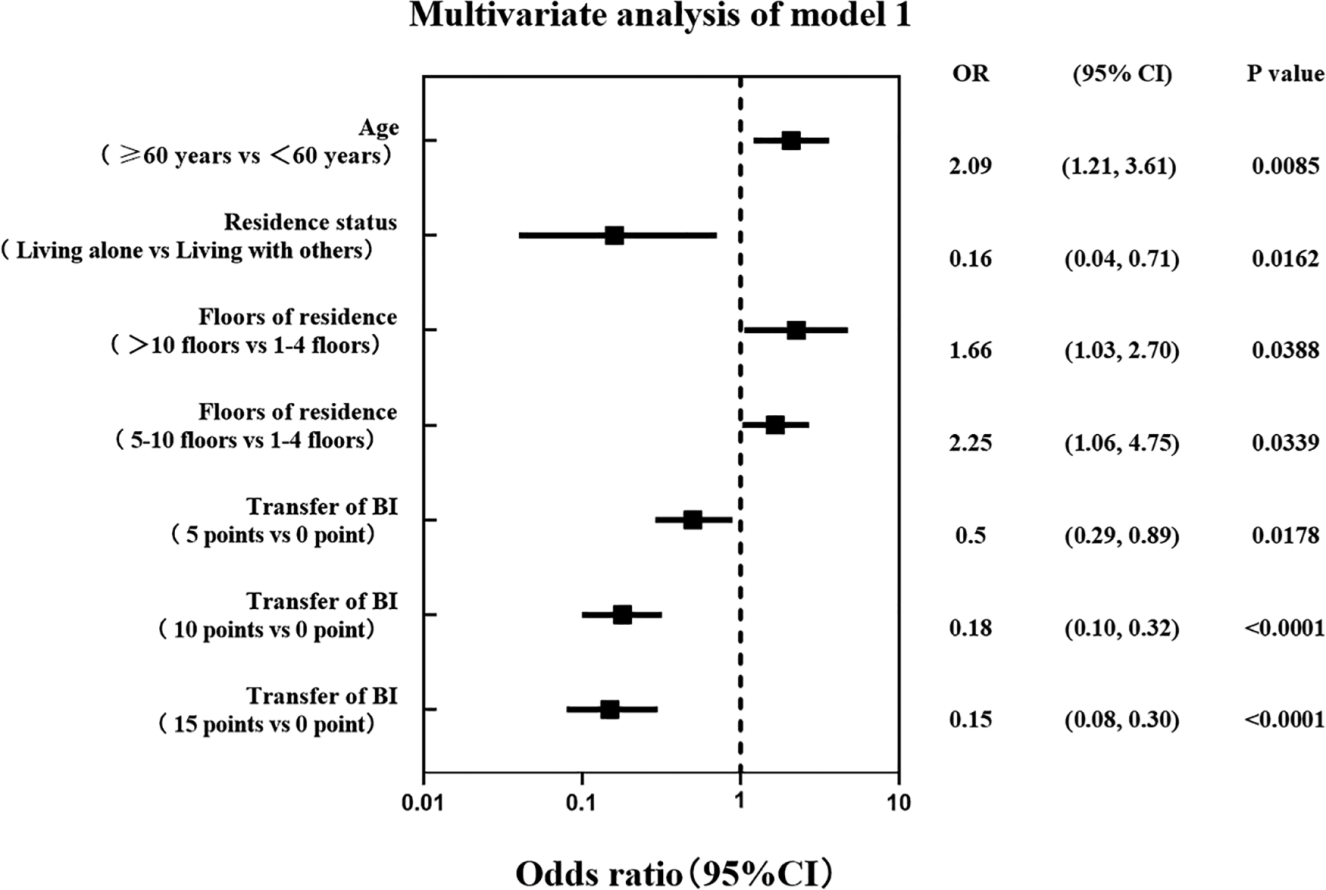
The multivariate analysis of model 1.

**Figure 5.**
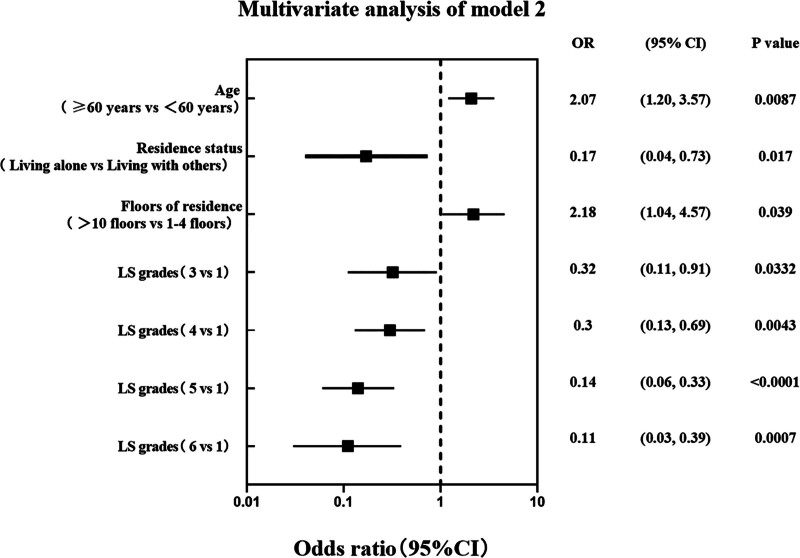
The multivariate analysis of model 2.

To further explore the factors influencing fall-related serious injury in patients with poststroke, we conducted a multivariate analysis of the factors which exhibited statistical differences in the univariate analysis (Figs. 4 and 5). Since both BI and LS are scales for evaluating ADL, we included each scale in separate models (Model 1 and Model 2) to avoid multicollinearity and to explore factors associated with fall-related serious injury from different perspectives. Model 1 showed that age, living situation, residential floor, and the BI’s bed-chair transfer item score were independent influencing factors for fall-related serious injury. Model 2 showed that age, living situation, residential floor, and LS grades were independent influences on fall-related serious injury.

In both Models, age was a risk factor for severe falls; patients aged over 60 years exhibited a higher risk of severe falls compared to those aged under 60 years (OR = 2.09, 95% CI: 1.21–3.61, *P* = .0085; OR = 2.07, 95% CI: 1.20–3.57, *P* = .0087). Residential floor was also a risk factor, with patients residing above the 10th floor facing 2.25- and 2.18-times higher risk in Models 1 and 2, respectively, compared to patients on lower floors (OR = 2.25, 95% CI: 1.06–4.75, *P* = .0339; OR = 2.18, 95% CI: 1.04–4.57, *P* = .0390). Notably, both models indicated a lower risk of fall-related serious injury in patients living alone (OR = 0.16, 95% CI: 0.04–0.71, *P* = .0162; OR = 0.17, 95% CI: 0.04–0.73, *P* = .0170).

In Model 1, the bed-chair transfer item score of the BI was identified as an independent protective factor. Patients who could complete this item independently or with assistance exhibited a lower risk of severe falls (OR = 0.50, 95% CI: 0.29–0.89, *P* = .0178; OR = 0.18, 95% CI: 0.10–0.32, *P* <.0001; OR = 0.15, 95% CI: 0.08–0.30, *P* <.0001; for scores 5, 10, and 15, respectively). In Model 2, the multivariate results were similar to the univariate results, indicating that grades 3 to 6 were protective factors against severe falls (OR = 0.32, 95% CI: 0.11–0.91, *P* = .0332; OR = 0.30, 95% CI: 0.13–0.69, *P* = .0043; OR = 0.14, 95% CI: 0.06–0.33, *P* <.0001; OR = 0.11, 95% CI: 0.03–0.39, *P* = .0007; for grades 3, 4, 5, and 6, respectively).

## 4. Discussion

Our study explored the factors influencing fall-related serious injury in patients with poststroke, considering the ADL perspectives. We found age, residential status, floor of the residence, BI’s bed-chair transfer item score, and LS grade as influencing factors on fall-related serious injury, as elucidated by 2 statistical models. These findings provide valuable insights for clinicians and nurses, emphasizing the importance of considering patients’ social participation and ADL in fall management, and facilitating the development of targeted interventions to more effectively prevent falls in patients with stroke.

In this study, we found that the incidence of serious injury among poststroke patients was as high as 20.19%, aligning with previous research findings. Prior studies have indicated that the incidence of serious injury following a stroke is approximately 30%, with a higher prevalence among patients with poorer functional status.^[11,28]^ Furthermore, Divani et al reported that 2 years poststroke, the incidence of subsequent injury and hip fracture due to falls was 15% and 2.1%, respectively.^[13]^ Compared to hospitalized patients, stroke patients exhibit a higher incidence of fall-related serious injuries. Previous research has shown that the likelihood of fall-related serious injury in hospitalized patients is generally below 10%.^[10,29]^ This discrepancy may be attributed to balance impairments, limb dysfunction, or cognitive deficits in stroke patients, which adversely affect their functional status and predispose them to fall-related injuries. Our findings also underscore the significance of bed-chair transfer ability, which is associated with the risk of fall-related injuries. Patients with compromised bed-chair transfer ability may have impaired balance or lower limb strength, increasing their susceptibility to severe fall-related injuries. This factor may aid clinicians and nurses in efficiently identifying stroke patients at risk for fall-related injury.

We found a positive correlation between age and fall-related serious injury, consistent with previous related study, which suggested that age is a risk factor influencing fall occurrence and fall-related injury.^[30]^ Previous studies have indicated that both community-dwelling and hospitalized elderly patients exhibit a higher incidence of falls and an increased subsequent risk of fall-related injuries.^[31–33]^ Given the disease characteristics of stroke, the balance and motor abilities may be impaired, making them more susceptible to fall-related serious injuries. Furthermore, although our model did not yield statistically significant results, the number of episodes remains noteworthy. Hardie et al observed that recurrent strokes may contribute to more severe disability, resulting in diminished functional levels.^[34]^ In general, patients with recurrent strokes tend to experience worse outcomes compared to those without recurrent strokes. Therefore, when patients with multiple recurrent strokes experience falls, the consequences may be more severe.

Our study revealed that both the residential floor of the home and living situation influence fall-related injury. In contrast, Tsai et al observed trends related to the impact of residential floor level on falls and the development of fear of falling among the elderly, but they did not achieve statistical significance.^[35]^ A plausible explanation for this disparity is the variation in study populations. Our study focused on patients with poststroke, who typically have lower functional independence compared to the elderly.^[22]^ Many poststroke patients have motor or cognitive disorders, further emphasizing the impact of their living situation on the occurrence of severe fall injuries. Notably, our study revealed reduced risk of severe fall-related injuries among patients with poststroke who lived alone. However, this result does not imply that living alone is preferable. Previous studies have shown that individuals living with others suffer higher frequency of falls.^[13]^ When regard to serious fall-related injury, patients living alone often demonstrate greater functional independence. Previous studies have shown that stroke patients with a functional independence measure scores >91 are capable of living independently.^[36]^ Therefore, stroke patients living alone may have a lower risk of experiencing severe fall-related events.^[37]^

Regarding ADL assessments, we observed that patients with better functional independence, as measured by both the BI and LS, had a lower risk of serious fall-related injury. Specifically, no significant difference existed in serious fall-related injury risk between completely and severely dependent patients. This may be explained by the fact that both groups exhibited lower ADL levels, which resulted in a higher risk of severe fall-related injuries for them. These findings are consistent with a previous study. Wei et al identified a positive correlation between fall incidence in patients with stroke and ADL, with higher functional independence measure scores associated with a reduced risk of falling.^[38]^ This suggests that when preventing falls and fall-related serious injuries, the ADL of poststroke patients, whether they reside in the community or are hospitalized, should be given particular consideration. Furthermore, previous studies have shown that both scales predict the risk of fall occurrence among stroke patients.^[11,39]^ Our study suggests that BI and LS may be useful in signaling the risk of serious fall-related injury in poststroke patients. In addition, using the BI and LS in separate models enables the identification of factors from different perspectives, facilitating a more comprehensive approach to fall management in stroke patients. Including the bed-chair transfer item from the BI in Model 1 helps capture the impact of balance ability and motor function on injurious falls. While using LS grades in Model 2 reflects differences in functional independence and activity range, which may also influence the risk of fall-related injuries.

Preventing falls has become an ongoing challenge for clinicians and nurses, particularly for patients with stroke who are functionally impaired.^[11,40]^ Although many fall prevention care plans are currently established, the problem of falls persists among patients with stroke. Therefore, shifting the focus towards identifying influencing factors to reduce fall-related serious injury is crucial. These factors must be carefully considered in the subsequent clinical management of falls. While focusing on the previously mentioned factors of mental status or disease characteristics on fall-related injury, the patient’s ADL is equally crucial. Patients with poor ADLs are at higher risk for falls and subsequent serious injuries, which may affect the functional recovery of patients with poststroke. While patients are undergoing rehabilitation, physicians and nurses should be alerted to the high risk of fall-related injuries in this group of hospitalized patients, and poststroke patients in the community should be also focused on education.

This study has some limitations. First, it did not collect data on other clinical risk factors that may influence fall-related serious injury, such as mental health issues or places to fall. The absence of this data may have affected our comprehensive evaluation of the factors contributing to fall-related serious injury. Second, this study solely utilized retrospective data from a cross-sectional study, preventing us from clarifying the causal relationship between factors and fall-related serious injury in patients with stroke. Further prospective research is required to establish causality.

## 5. Conclusion

In this study, we found 2 statistical models to identify factors influencing fall-related serious injury in patients with poststroke, which included age, living situation, residential floor, the BI’s bed-chair transfer item scores, and LS grading. These findings provide a reference for implementing effective fall management and prevention measures for patients with stroke.

## Acknowledgments

We would like to thank all patients who participated in this study and all medical staff who participated in data collection. We also would like to thank Editage (www.editage.cn) for English language editing.

## Author contributions

**Conceptualization:** Yucong Zou, Fubing Zha, Jing Zhou, Yulong Wang.

**Formal analysis:** Yucong Zou, Fubing Zha.

**Methodology:** Weiming Lin.

**Resources:** Weiming Lin.

**Writing – original draft:** Yucong Zou.

**Writing – review & editing:** Fubing Zha, Jing Zhou, Yulong Wang.
